# A rare case of DRESS (Drug Reaction With Eosinophilia And Systemic Symptoms) with important involvement of heart, liver, central nervous system and bone marrow

**DOI:** 10.1186/2045-7022-4-S3-P19

**Published:** 2014-07-18

**Authors:** Jan Schroeder, Antonella Citterio, Gloria Anversano, Ambra Mascheri, Edgardo Bonacina, Olivia Leoni, Giuseppe Scibilia, Alberto Roghi, Chiara Gamba, Elide Pastorello

**Affiliations:** 1 Niguarda Hospital, Italy; 2 Niguarda Hospital, Burn Unit, Italy; 3 Niguarda Hospital, Allergy and Immunology Unit, Italy; 4 Niguarda Hospital, Pathology Service, Italy; 5 Niguarda Hospital, CMR Unit, Department of Cardiology, Italy; 6 Ospedale Riuniti, GISED, Italy

## 

A 46 year old woman from Sri Lanka, underwent blood examination after her return to Italy and found high titers of uric acid (8.2 mg/dl), thus her family physician prescribed allopurinol 300 mg once daily. After 3 weeks she presented to the ER with fever and esantematic rash on the thorax and limbs, therefore allopurinol was stopped and began prednisone 25 mg/day. A week later she returned to the ER for a worsening of the cutaneous eruption, high fever and marked weakness. The admitting physician excluded an adverse drug reaction and was admitted to the Infectious diseases ward with altered liver function tests, high inflammation indices and eosinophilia. All the tests for infectious disease workup resulted negative, but her general condition worsened with high fever, anasarcatic status and neurological weakness. The total body CT scan showed lung and abdominal lymphadenopathy and pleural effusion. The bone marrow biopsy excluded a lympho myeloproliferative disorder and revealed two microgranulomas and 34% eosinophils. The hepatic biopsy showed a diffuse acute hepatitis with marked infiltrate of T-lymphocytes (CD3+) and eosinophils. Cardiac examination with EKG and echocardiography was normal but the cardiac MNR dimostrated a severe pericardial inflammation with effusion. Only when the eosinophilic blood concentration raised to 52% the diagnosis of DRESS was made. The patient also resulted positive for HLA-B*58:01. She began treatment with high glucocorticoid i.v. (1 mg/kg/day) and obtained rapid improvement of the general condition of weakness, skin healing by desquamation and normalization of eosinophilia and hepatic function. She was discharged after one month of hospital stay.

## Conclusion

This is a particular case of DRESS with extensive histological, blood, and radiological examination. It is important to employ elevated levels of glucocorticoids in the treatment of DRESS. The normal cardiac screening by EKG and echocardiography may sometimes prove inadequate for identifying real cardiac injury. We believe that it's important to undergo cardiac MR imaging since appropriate therapy may prevent progression of cardiac disease.

